# Highlighting the importance of biodiversity conservation through the Holy Qur'an

**DOI:** 10.1111/cobi.14309

**Published:** 2024-06-06

**Authors:** Lisa A. Blankinship, Sarah Gillaspie, Basil H. Aboul‐Enein

**Affiliations:** ^1^ Department of Biology University of North Alabama Florence Alabama USA; ^2^ Department of Family Medicine, School of Medicine Virginia Commonwealth University Richmond Virginia USA; ^3^ Faculty of Public Health and Policy London School of Hygiene & Tropical Medicine London UK

**Keywords:** biodiversity, conservation, Holy Qur'an, Islam, sustainable development goals, biodiversidad, conservación, islam, objetivos de desarrollo sustentable, Sagrado Corán

## Abstract

Religious environmentalism relies upon religious texts and leadership to promote effective and long‐lasting change for environmental problems, such as responsible use and conservation of natural resources and biodiversity. World religions note the importance of biodiversity and humanity's responsibility in stewarding biodiversity as a member of ecological communities. We reviewed Quranic verses that relate to biodiversity and align with United Nations Sustainable Development Goals (SDGs). The Holy Quran was reviewed in electronic and hard copy formats, and verses related to biodiversity were translated to English and tabulated by Qur'anic chapter, verse, and narrative citation. Twenty‐one Qur'anic verses were identified that addressed biodiversity. Scriptures were divided into 5 groups that addressed provision of resources, governance or stewardship of resources, nature as a teacher, and human life in nature's communities or described creation of biodiversity. Qur'anic verses were aligned with 4 SDGs (goals 12–15), which address sustainable consumption of natural resources, global climate change, life in marine environments, and life in terrestrial environments, including freshwater ecosystems. This alignment demonstrates the interconnectedness of life, that conservation of biodiversity is referenced in the Quran, and how positive management of natural recourses can be beneficial to Muslim communities on local, national, and global scales. Positive movement toward ecofriendly practices, sound environmental resource use and management, biodiversity conservation, and governmental policies on conservation can be promoted through scriptures from the Holy Qur'an.

## Abstract (es)

Énfasis en la importancia de la conservación de la biodiversidad con el Sagrado Corán

**Resumen:**

El ambientalismo religioso depende de los textos y el liderazgo religioso para promover un cambio efectivo y duradero de los problemas ambientales, como el uso y conservación de los recursos naturales y la biodiversidad. Las religiones del mundo destacan la importancia de la biodiversidad y la responsabilidad de la humanidad en el cuidado de la biodiversidad como miembros de las comunidades ecológicas. Revisamos los versos del Corán que se relacionan con la biodiversidad y que se alinean con los Objetivos de Desarrollo Sustentable (ODGs) de las Naciones Unidas. Revisamos el Sagrado Corán en formato físico y electrónico y tradujimos al inglés los versos relacionados con la biodiversidad para luego tabularlos según la cita del capítulo, verso y narrativa del Corán. Identificamos 21 versos del Corán en los que se aborda la biodiversidad. Clasificamos las escrituras en cinco grupos de acuerdo con si abordan el suministro de recursos, la administración o cuidado de los recursos, a la naturaleza como docente y a la vida humana dentro de las comunidades naturales o si describen la creación de la biodiversidad. Los versos del Corán se alinearon con cuatro de los ODGs (del 12 al 15), lo cuales abordan el consumo sustentable de los recursos naturales, el cambio climático mundial y la vida marina y terrestre, incluyendo los ecosistemas de agua dulce. Esta alineación demuestra la interconexión de la vida, que el Corán tiene referencias a la conservación de la biodiversidad y cómo la gestión positiva de los recursos naturales puede ser benéfica para las comunidades musulmanes a escala local, nacional y mundial. El Sagrado Corán puede usarse para promover un movimiento positivo hacia las prácticas amigables con el ambiente, el uso y manejo sensato de los recursos naturales, la conservación de la biodiversidad y políticas gubernamentales para la conservación.

## INTRODUCTION

Religious environmentalism is a perspective that emphasizes the interconnectedness of religious beliefs, ethics, and practices (Kula, 2001). Gottlieb (2006) notes that religion is a powerful voice in correcting, eliminating, and improving ecological problems through recognition of how environmental resources are best used to support all people in a community and in promoting the value of creation (humans and other organisms). It is this recognition of moral and spiritual responsibilities of individuals and communities to protect and preserve the natural world as an integral part of religious practice that is diametrically opposed to the prevailing thought of the late 19th and early 20th centuries that focused on science and technology as a means to solve all humanity's problems (Ozdemir, 2003). Ozdemir (2003) further notes that due to the societal shift in emphasizing science and technology rather than religion, society has lost a facet of their spirituality and no longer viewed conservation as a religious mandate. Religious environmentalism seeks to leverage religious texts and practices to promote behavior change that leads to environmental conservation and advocacy (Gottlieb, 2006).

Religious environmentalism finds expression in faith traditions worldwide, including Islam, Christianity, Judaism, Hinduism, Buddhism, and others (Adriance et al., 2010). Eastern religions are intrinsically more aligned with balance and environmental conservation than religions that trace their heritage to Abraham (Tomalin, 2016). However, our emphasis is on religious environmentalism in the Islamic faith and how the Qur'an supports the United Nation's Sustainable Development Goals (UN SDGs).

Religious environmentalism acknowledges that Earth and its ecosystems are sacred and worthy of reverence. It emphasizes that the natural world is a manifestation of the divine and therefore should be treated with care and respect (Kula, 2001; Sadowski & Ayvaz, 2023). This perspective encourages believers to view environmental issues, such as biodiversity conservation, not only as matters of practical importance but also as moral and spiritual concerns. Religious environmentalism recognizes humans as stewards of Earth (Aboul‐Enein, 2018; Saniotis, 2012). It emphasizes the responsibility to well manage and protect the environment, including its resources, such as water, air, land, and biodiversity. Believers are called upon to act as responsible caretakers and thus to ensure the sustainable use of resources and the preservation of ecosystems for current and future generations (Anthwal et al., 2010; Berkes, 2013; Skórka et al., 2018).

Religious environmentalism often incorporates ethical principles in environmental decision‐making (Koehrsen, 2021; Laxman et al., 2014; Sadowski & Ayvaz, 2023). These principles may include concepts such as justice, compassion, intergenerational equity, and the well‐being of all creation (Irawan, 2022; Jusoff & Abu Samah, 2011; McKay et al., 2014). Such ethical considerations guide believers to make choices that promote environmental sustainability, conservation, and social justice and recognize the interconnectedness of all living beings. As such, religious environmentalism encourages education and awareness about environmental issues within religious communities. It promotes initiatives that highlight the importance of sustainable practices, conservation, and environmental justice. For believers in developing counties, practicing conservation may be socioeconomically prohibitive, regardless of personal faith and encouragement from religious leadership (Tomalin, 2016). Academic studies have long overlooked the influence of religion on personal and community relationships with nature, including resource acquisition and management (Tomalin, 2016).

Religious leaders and organizations often engage in advocacy efforts to address ecological challenges and promote policies aligned with environmental stewardship (Bhagwat et al., 2011; Irawan, 2022; Koehrsen, 2021). Religious organizations can affect changes for environmental causes through the dissemination of proenvironmental values and worldviews to their constituencies and thereby influence lifestyles or advocate for needed change (Koehrsen, 2021; Tucker & Grim, 2001). Many religious traditions incorporate rituals and practices that express reverence for the natural world and promote environmental awareness, including ceremonies to bless and protect natural resources, prayers for the well‐being of Earth, and practices that encourage simplicity, frugality, and mindful consumption (Jusoff & Abu Samah, 2011; Koehrsen, 2021; Turnbull, 2021). For example, an Islamic Indonesian boarding school teaches environmental protection practices and a Ramadan *iftar* (fast‐breaking evening meal) favoring locally grown produce to break the daily fasts of Ramadan (Bratton, 2018). These teachings exemplify *pesantren Ath‐Taariq* (the way).

Biodiversity conservation and religious environmentalism are integral to meeting several of the UN SDGs, including climate action; conservation and sustainable use of oceans, seas, and marine resources (life below water); and protection, restoration, and promotion of sustainable use of terrestrial ecosystems (life on land) (UNDESA, 2023). Meeting these goals requires people to change their behavior so as to support sustained environmental conservation. Such behavioral changes could be further promoted and supported by religious community buy‐in (Gade, 2012). Ideally, commitment to conservation would become a life habit such that believers continue conservation efforts even during hardships, such as those observed during the COVID‐19 pandemic. Progress toward the SDGs was either halted or negatively affected due to the pandemic (Yuan et al., 2023). While the consumption of natural resources may have decreased, so too did technology and policies that promoted climate action, life below water, and life on land. Progress toward the remaining SDGs was also negatively affected by the pandemic and in the years following (Yuan et al., 2023).

We aimed to highlight verses found in the Holy Qur'an that relate to the conservation of biodiversity. Religious leaders, environmental advocates, and policy makers can utilize religious environmentalism to encourage acceptance of policies and needed behavioral changes within their faith communities to help meet the SDGs, specifically those related to climate action, life below water, and life on land. By integrating environmental concerns into religious beliefs and practices, individuals and communities are encouraged to work toward a more harmonious relationship with the natural world and embrace a holistic approach that recognizes the spiritual, ethical, and practical dimensions of environmental stewardship and biodiversity conservation.

## REVIEW OF THE QURAN

We followed similar methodological search parameters outlined in previously published studies relevant to Qur'anic studies research and environmental health (Aboul‐Enein, 2018; Aboul‐Enein et al., 2024). The Holy Qur'an was comprehensively reviewed in electronic and hard copy formats (The Holy Qur'an, 2023; The Noble Qur'an, 1993). Both formats were reviewed in English and Arabic to identify themes and content connected to concepts and practices and behavioral guidelines as they relate to the importance of biodiversity and related concepts. All relevant contents were translated to English and then tabulated by Qur'anic chapter, verse, and narrative citation (Table 1). The King Fahd Complex for the Printing of the Holy Qur'an and the King Saud University Electronic Moshaf Project provided English translations for the purpose of this review (Aboul‐Enein, 2018; Aboul‐Enein et al., 2024). For the purposes of this review, the *Hadith* (sayings of the Prophet Muhammed) and *Sunnah* (traditions and practices of the Prophet Muhammed) were excluded.

**TABLE 1:** Qur'anic verses highlighting the importance biodiversity.

Chapter	Place of revelation	Citation	Verse quotation
Surat An‐Naml (The Ant or The Ants)	Mecca	27:60	Is He [not best] who created the heavens and the earth and sent down for you water from the sky, causing to grow thereby gardens of joyful beauty and delight which you could not [otherwise] have grown the trees thereof?
Surat Al‐Hashr (The Exile, The Banishment, The Mustering, or The Gathering)	Medina	59:5	Whatever palm‐tree ye cut down or leave standing upon its roots, is only in accordance with His will. For God will surely humiliate such wickedness.
Surat Al‐An'am (The Grazing Livestock or The Cattle)	Mecca	6:99	And it is He who sends down rain from the sky, and with it We bring forth diverse vegetation of all things. We produce from it green stalks from which We produce grains arranged in layers. And from the palm trees—of its emerging fruit are clusters hanging low. And [We produce] gardens of grapevines, olives and pomegranates, similar yet different (in diversity). Look at their fruits when it yields and [at] its ripeness. Indeed! In these things, there are signs for people who believe.
Surat Al‐Rahman (The Most Merciful or The Most Gracious)	Medina	55:10	And the earth He has assigned for all living creatures.
Surat At‐Tariq (The Night comer or The Morning Star)	Mecca	86:12	By the sky which gives rain again, and the earth which splitteth (with the growth of trees and plants).
Surat An‐Nahl (The Honey Bees)	Mecca	16:13	And whatsoever He has created for you on this earth of varying colors [and qualities from vegetation and fruits (botanical life) and from animal (zoological life)]. Verily! In this is a sign for people who remember.
Surat Al‐An'am (The Grazing Livestock or The Cattle)	Mecca	6:38	There is not a moving (living) creature on earth, nor bird that flies with its 2 wings, but (forms part of) communities like you.
Surat Al‐Luq'man (Luqman the Wise)	Mecca	31:10	He has created the heavens without any pillars that you see, and has set on the earth firm mountains, lest it should shake with you. And He has scattered therein moving (living) creatures of all kinds. And We send down water (rain) from the sky, and We cause (plants) of every goodly kind to grow therein.
Surat An‐Nahl (The Honey Bees)	Mecca	16:11	With it (the rain) He brings up for you the crops, olives, dates, the grapes and every kind of fruit. Verily! In this is indeed evidence and manifest sign for people who give it thought.
Surat Al‐An'am (The Grazing Livestock or The Cattle)	Mecca	6:141	And it is He Who produced gardens, both trellised and untrellised, and date palms, and crops of different shape and taste (their fruits and their seeds) and olives, and pomegranates, similar (in kind) and different (in taste). Eat of the fruits when they ripen. But be not excessive. Indeed, He does not like those who commit excess.
Surat Ar‐Ra'd (The Thunder)	Medina	13:3–4	And it is He who spread out the earth, placed therein firm mountains and rivers and variety of fruits (in pairs) on it. He brings the night as a cover over the day. Verily, in these things, there are lessons for people who reflect. And on the earth are neighboring tracts, gardens (vineyards), cultivated green fields, and date palms growing diversely, watered from a single source, yet we make some of them (fruits) exceed (quality of) in food value to eat. Behold, verily in these things there are signs for those who use their reason.
Surat Al‐Baqarah (The Cow or The Heifer)	Medina	2:266	Would any of you wish to have a garden full of date palms and vines [grapes] through which rivers flow underneath? He would have all sorts of fruits in it…
Surat Al‐Kahf (The Cave)	Mecca	18:45	And put forward to them the example of the life of this world, it is like the water (rain) which We send down from the sky, and the vegetation of the earth mingles with it, and becomes fresh and green…
Surat Ta‐Ha (The Letters T–H)	Mecca	20:53–54	Who has made earth for you like a bed (spread out); and has opened roads (ways and paths) for you therein; and has sent down water from the sky. And We have brought forth with it various kinds of vegetation. Eat and pasture your cattle; Indeed! In this are proofs and signs for men of understanding.
Surat Al‐Qamar (The Moon)	Mecca	54:49	Verily, all things have We created in proportion and measure.
Surat Al‐Rahman (The Most Merciful)	Medina	55:7–9	And the sky has He raised high, and has devised (for all things) a balance, so that you might never transgress the balance: weigh, therefore with equity, and do not upset the balance.
Surat Al‐Hijr (The Rocky Tract or The Stoneland)	Mecca	15:19	And the earth—We have spread it and cast therein firmly set mountains and caused to grow therein all kinds of every well‐balanced thing (in due proportion).
Surat Ash‐Shura (The Consultation or The Council)	Mecca	42:27	And if He had extended excessive abundance for His servants, they would behave with insolence throughout the earth. But He sends [it] down in amounts (due measure) which He wills. For, verily, He is fully aware [acquainted] of [the needs of] His creatures.
Surat Al‐A'raf (The Heights)	Mecca	7:56	And cause not corruption upon the earth after it hath been set in order…
Surat Al‐A'raf (The Heights or The Elevation)	Mecca	7:85	…And do not spread corruption on earth after it has been so well ordered: [all] this is for your own good, that will be best for you if ye have faith.
Surat An‐Nur (The Light)	Medina	24:45	God has created every [living] creature from water. And of them are those that move on their bellies, and of them are those that walk on 2 legs, and of them are those that walk on four…

## QURANIC VERSES RELATED TO BIODIVERSITY

Twenty‐one Qur'anic verses were identified that specified biodiversity in the created world. Of these 21 verses, 15 were revealed in Mecca and 6 in Medina. Fourteen of the 21 verses reference a terrestrial environment, whereas one verse focused on the aquatic environment as being key to creation. Fourteen verses address provision of rain, sun, and other factors that lead to plant and animal life (Al‐An'am 6:99 and 6:141; Ar‐Ra'd 13:3–4; Al‐Hijr 15:19; An‐Nahl 16:11 and 16:13; Al‐Kahf 18:45; Ta‐Ha 20:53–54; An‐Naml 27:60; Al‐Luq'man 31:10; Ash‐Shura 42:27; Al‐Qamar 54:49; and Al‐Rahman 55:7–9 and 55:10). Seven verses encourage governance or management of resources (Al‐Baqarah 2:266; Al‐An‐am 6:141; Al‐A'raf 7:56 and 7:85; Ta‐Ha 20:53–54; Al‐Rahman 55:7–9; and Al‐Hashr 59:5). Eleven verses directly address biodiversity (Al‐Baqarah 2:266; Al‐An‐am 6:38, 6:99, and 6:141; Ar‐Ra'd 13:3–4; An‐Nahl 16:11 and 16:13; Ta‐Ha 20:53–54; An‐Nur 24:45; Al‐Luq'man 31:10; and At‐Tariq 86:12). Five references recognizing all life as constituting a community and encouraging balance in that community (Al‐An'am 6:38; Al‐A'raf 7:56 and 7:85; Al‐Hijr 15:19; and Al‐Rahman 55:7–9). Two verses point to nature as a source of instruction (Ar‐Ra'd 13:3–4 and Al‐Kahf 18:45).

## THE QURAN, CONSERVATION, AND SDGs

Qur'anic verses discuss biodiversity as originating from the divine and being entrusted to humanity as members of a living community; thus, good stewardship of biodiversity falls within scriptural guidance (Table 1). Ecology, as noted by Azizy et al. (2024), can be viewed as the interrelated activities between humans and the environment, a mutualistic relationship between the various aspects of creation. In their review of verses from Al‐Baqarah, Azizy et al. (2024) note that God appointed humans as God's representative on Earth and it is humanity's responsibility to manage what was created. Suwar Ta‐Ha 20:53–54 and Al‐Rahman 55:10 provide further instruction for good ecological management, and surat Al‐A'raf 7:56 and 7:85 caution against corrupting the earth.

Conservation of biodiversity is also supported by the UN SDGs. Goal 14 focuses on sustainability and conservation of the marine environment, and goal 15 focuses on terrestrial ecosystems (UNDESA, 2024a). Because freshwater ecosystems are tightly linked to and influenced by terrestrial environments, they are also covered in goal 15 (UNDESA, 2024a). Response to climate change is supported by goal 13 and is applicable to discussions of diversity because climate change influences biological zones and thus biological processes often affect biological community structure in specific geographic areas. Additionally, ecotourism (goal 12) promotes education and sustainability (UNDESA, 2024a).

Goal 14 focuses on the conservation and sustainable development of marine environments (UNDESA, 2024a). In surat An‐Nur 24:45, the importance of water is noted in the creation of all creatures. Because life was created by God, it has value and thus should be well managed. Although freshwater ecosystems are addressed by goal 15, water is still essential to survival in terrestrial environments for hydration, production of food, biogeochemical cycling, and acquisition of nutrients. Marine ecosystems are key to global environmental functioning, represent the largest carbon sink, and are a significant source for oxygen production via phytoplankton and cyanobacteria (National Oceanic & Atmospheric Administration National Ocean Service, 2023; United Nations Climate Action, 2024). As with terrestrial environments, life in the oceans is diverse. Oceans host over 240,000 known species as of a 2021 census and contain an estimated 1.4–1.6 million species (World Register of Marine Species, 2024). Freshwater environments are estimated to contain approximately 140,000 species (World Wide Fund for Nature, 2024b). When counting species, estimates may not reflect the true number of unique species in an environment due to differences in sampling methods. Maldonado et al. (2015) suggest that the use of big data, such as data from polymerase chain reaction and genomic sequencing, may provide a higher estimate than actual species counts. However, polymerase chain reaction and genomic sequencing are key to accurate counts of low‐density species in aquatic environments because aquatic environments often present a challenge for microorganism culture, which means accuracy of organism counts may be skewed with traditional culture‐based enumeration methodologies (Nathan et al. (2014). As with terrestrial environments, organisms are not equally distributed within the water column.

Goal 14.1 (Table 2) addresses ocean and sea health though the reduction of plastic debris and nutrient runoff, which would produce overgrowth of microbes and thus lead to overuse of oxygen and fish kill. Islam and Samsudin (2018) address the impacts of eutrophication events, such as red tides or harmful algal blooms (HABs) from the perspective of the Qur'an, noting that HABs are the direct result and responsibility of humans. An HAB can additionally be viewed as human contamination of the environment, as mentioned in surat Al‐A'raf 7:56 and 7:85. An‐Nur 24:45 notes the key role water holds as the source of creation. Additionally, aquatic environments are important to biogeochemical cycling and food production; thus, marine environments should be sustainably managed. Bsoul et al. (2022) state that both the Qur'an and Prophet Muhammad promote environmental sustainability on a community and personal level and note the importance of education to promote environmental sustainability behaviors.

**TABLE 2:** Conservation targets relevant to Holy Qur'an verses that align with United Nations sustainable development goals (SDGs) 12–15.

SDG target	Stated objective
14.1	By 2025, prevent and significantly reduce marine pollution of all kinds, in particular from land‐based activities, including marine debris and nutrient pollution.
14.3	Minimize and address the impacts of ocean acidification, including through enhanced scientific cooperation at all levels.
14.7	By 2030, increase the economic benefits to Small Island developing States and least developed countries from the sustainable use of marine resources, including through sustainable management of fisheries, aquaculture and tourism.
15.1	By 2020, ensure the conservation, restoration and sustainable use of terrestrial and inland freshwater ecosystems and their services, in particular forests, wetlands, mountains and drylands, in line with obligations under international agreements.
15.2	By 2020, promote the implementation of sustainable management of all types of forests, halt deforestation, restore degraded forests and substantially increase afforestation and reforestation globally.
15.3	By 2030, combat desertification, restore degraded land and soil, including land affected by desertification, drought and floods, and strive to achieve a land degradation‐neutral world.
15.4	By 2030, ensure the conservation of mountain ecosystems, including their biodiversity, in order to enhance their capacity to provide benefits that are essential for sustainable development.
15.5	Take urgent and significant action to reduce the degradation of natural habitats, halt the loss of biodiversity and, by 2020, protect and prevent the extinction of threatened species.
15.6	Promote fair and equitable sharing of the benefits arising from the utilization of genetic resources and promote appropriate access to such resources, as internationally agreed.
13.2	Integrate climate change measures into national policies, strategies and planning.
13.3	Improve education, awareness‐raising and human and institutional capacity on climate change mitigation, adaptation, impact reduction and early warning.
12.b	Develop and implement tools to monitor sustainable development impacts for sustainable tourism that creates jobs and promotes local culture and products.

Goal 14.3 seeks to reduce ocean acidification (Table 2). Although surat Al‐An'am 6:38 addresses community structure inclusive of animals in a terrestrial environment, often actions of community members on land will affect marine communities, as exemplified by ocean acidifications. The overproduction of CO_2_ leads to acidification of the oceans, which causes ocean chemistry to shift and decreases the availability of nutrients required by crustaceans for exoskeleton development in addition to directly limiting marine life, such as coral. A primary source for ocean acidification is the production of CO_2_ emissions from industrial applications. Changing to more sustainable energy and transportation methods will help offset CO_2_ emissions, and development of carbon‐capturing and sequestration methodologies has been suggested for excess atmospheric CO_2_ removal (Madejski et al., 2022). Shaari et al. (2020) reviewed CO_2_ emissions from oil and gas consumption and recommend that, although other energy sources should be considered, energy production from oil and gas sources can be improved to help offset pollution. The type of energy source in addition to the amount of energy being used should be considered when looking at sustainable and environmentally appropriate methodologies for energy production and use, especially for nations that produce large amounts of petroleum‐based energy sources (Shaari et al., 2020).

Goal 15 focuses on the conservation and sustainable development and use of terrestrial environments primarily through protection of ecosystems, management of terrestrial resources in light of climate change, and conservation of biodiversity (UNDESA, 2024a). Several Qur'anic verses speak to biodiversity in terrestrial environments: Al‐Baqarah 2:266; Al‐An'am 6:38, 6:99, and 6:141; Ar‐Ra'd 13:3–4; Al‐Hijr 15:19; An‐Nahl 16:11 and 16:13; Al‐Kahf 18:45; Ta‐Ha 20:53–54; An‐Naml 27:60; Al‐Luq'man 31:10; Al‐Hashr 59:5; and At‐Tariq 86:12. The most common motif in these verses is a flourishing garden—a description of what was entrusted to humans and what humanity should seek to promote and conserve (Azizy et al., 2024). Surat Ar‐Ra'd 13:3–4 describes the garden (Earth) as it was originally entrusted to humanity—beautiful, fertile, and useful for cultivation. Al‐Baqarah 2:266 questions whether anyone would like such a garden as is described in Ar‐Ra'd 13:3–4, which implies that nature, as it was originally entrusted to humanity, can serve as a teacher for how to maintain such a garden. The UN groups freshwater ecosystems with the terrestrial environment; thus, surat An‐Nur 24:45 would also be applicable to this SDG.

The Qur'an includes several verses that note or describe the types of biodiversity created, but there are few verses (e.g., Suwar Al‐A'raf 7:56 and 7:85; Ash‐Shura 42:27; Al‐Hashr 59:5) that specifically address the importance of biodiversity management or charge people to manage natural resources. However, Idami et al. (2022) note that throughout the history of Islam, doing good deeds is a key practice and teaching, which can be applied to deeds to other creatures, such as plants and animals. Idami et al. (2022) also note that many individuals see nature and what nature can provide for them personally rather than how they can protect and preserve nature for the good of all. When community members are educated about the benefits of respecting biodiversity and how to preserve it, they are more likely to take action and positively govern the resources available to them. Bsoul et al. (2022) note that religious motivation can be a powerful incentive for changing behavior patterns so that individuals would distinguish among what is truly needed, what is wanted, and what is in excess of need.

The management of natural resources, whether personally, at the community level, or at the state level, is encouraged by Suwar Al‐A'raf 7:56 and 7:85 and Al‐Rahman 55:7–10. Good stewardship of plant and animal biodiversity is noted in goals 15.1–15.6 (Table 2). Each of the target goals focuses on a different part of the natural world: 15.1 emphasizes conservation of freshwater ecosystems, 15.2 focuses on forests conservation, 15.3 seeks to limit and reverse desertification, 15.4 looks to preserve mountain ecosystems, 15.5 links habitat degradation and species extinction, and 15.6 promotes sharing of resources (UNDESA, 2024c).

Goal 13 relates to habitat management and focuses on how climate change can be stopped (UNDESA, 2024b). As global climate change continues, decreases in biodiversity and population shifts are expected to occur as organisms adjust or fail to adjust to their changing environment (Bellard et al., 2012). Natural events, such as the El Niño Southern Oscillation and volcanic eruptions, are contributing to this global temperature rise (Bethke et al., 2017; McPhaden et al., 2020). However, these events will continue to contribute to the consequences of global climate change, including changes in precipitation patterns, intensification of storms, and increased sea level rise (World Wide Fund for Nature, 2024a). Effective management strategies will promote education of individuals, groups, law makers, and governmental leaders (goal 13.3) as well as regional and national policies that promote conservation efforts (goal 13.2). Suwar Al‐A'raf 7:56 and 7:85, Ta‐Ha 20:53–54, Al‐Rahman 55:7–10, and Al‐Hashr 59:5 support goals 13.2 and 13.3 (Table 2). Surat Al‐A'raf 7:56 and 7:85 advocate for not contaminating or polluting the environment. Education, policy changes, strategies, and plans that move toward pollution reduction and decreases in climate change would be examples of how this surat can be used to promote actions in favor of goal 13.

Marine and terrestrial environments are interconnected through biogeochemical cycles, nutrient acquisition processes, and food production, so often what affects one environment affects another. Surat Al‐Baqarah 2:266 notes this interaction between water and land—it is the rivers that flow beneath the garden that provide nutrients and water for the flourishing garden. Finzi et al. (2011) note that temperature changes influence gas states of key elements, such as carbon and nitrogen, and thus limit microbial and plant fixation of these elements in terrestrial environment and suggest that similar impacts would occur in aquatic environments. Similarly, bacteria that play key roles in the nitrogen cycle by breaking down nitrogen compounds and converting them to bioactive molecules other organisms can use have an optimal growth temperature range of 15–32°C (Zhang et al., 2019). As temperatures shift due to climate change, this affects microbial communities and plant species that are key in the conversion of geochemical materials into forms useable by other organisms. Disruption of these cycles would have detrimental impacts for the affected areas and potentially disrupt food and resource acquisition across multiple geographic areas.

Ecotourism is a rapidly growing field that supports 10% global tourism jobs and gross domestic product (Ver Steeg, 2022). Sustainable tourism is supported by goal 12.b (Table 2). Ver Steeg (2022) note that although traditional tourism is primarily concerned with economic growth and mass influxes of tourists, ecotourism is concerned with sustainable development, education, and conservation. Surat Al‐A'raf 7:56 and 7:85 caution against corruption or pollution that often comes with the rapid overdevelopment associated with tourism. Ecotourism seeks sustainable development that will not overtax land and water resources or cause unnecessary pollution. In the Middle East, Oman and Jordan have successfully developed ecotourism options that help the national economy and provide jobs for public and private tourism industry. Ver Steeg (2022) reviews the challenges and successes of Jordan's and Oman's ecotourism industry.

## CONCLUSION

We found that 21 Quranic verses address biodiversity, management of natural resources, the ecological community and humanity's responsibility to it, nature as a teacher, and God as providing the necessary resources for creation. Verses from the Holy Quran align with 4 of the UN's sustainability goals, including responsible consumption and production (goal 12), climate action (13), and goals associated with life below water (14) and life on land (15). The identified verses can be utilized by religious leaders, environmental advocates, and policy makers to promote education and advocate for behavior changes that could increase conservation efforts and help to meet the SDGs by 2030. Although individual actions are a personal choice, members of religious communities are more likely to make sustainable action changes if there is a connection to their faith. Although we focused solely on the Holy Qur'an and the Islamic faith, other world religions provide guidance for how humans should interact with and steward natural resources, including biodiversity. As such, holy texts from other major world religions provide a valuable resource for future research in the field of religious environmentalism, especially if religious texts can be aligned with the UN SDGs and promote actions to meet the 2030 agenda. For example, a review of the Holy Bible and the Christian faith may provide motivation for more responsible consumption among Christians. Additionally, research into personal choices based on religious guidance would provide useful information as to the effectiveness of religious environmentalism. An interesting research question is what the impact of religious guidance on use of sustainable energy, recycling of plastics, and selection of long‐term‐use materials in lieu of single‐use plastics is.
